# Specificity and Selectivity of Raman Spectroscopy for the Detection of Dose‐Dependent Heavy Metal Toxicities

**DOI:** 10.1002/pld3.70086

**Published:** 2025-06-23

**Authors:** Isaac D. Juárez, Nicholas Shepard, Cole Sebok, Sudip Biswas, Endang Septiningsih, Dmitry Kurouski

**Affiliations:** ^1^ Department of Biochemistry and Biophysics Texas A&M University College Station Texas USA; ^2^ Interdisciplinary Faculty of Toxicology Texas A&M University College Station Texas USA; ^3^ Interdisciplinary Graduate Program in Molecular & Environmental Sciences Texas A&M University College Station Texas USA; ^4^ Department of Biology Texas A&M University College Station Texas USA; ^5^ Department of Soil and Crop Sciences Texas A&M University College Station Texas USA

**Keywords:** 2D‐COS, arsenic, cadmium, dose–response, ICP‐MS, lead, *Oryza Sativa*, PLS‐DA, vibrational spectroscopy

## Abstract

Contamination of farmland with heavy metals (HMs), particularly arsenic, cadmium, and lead, poses significant risks to human health and food security, especially through HM bioaccumulation in rice ( *Oryza Sativa* ). Current methods of detection for HMs, such as ICP‐MS, provide accurate measurements but are destructive and labor‐intensive, limiting their feasibility for widespread agricultural use. In this study, we investigated the potential of Raman spectroscopy (RS) as a nondestructive, cost‐effective alternative for the detection of HM stress and thereby uptake in rice. Using a dose–response experimental design, we examined the sensitivity of RS for detecting varying levels of arsenic, cadmium, and lead‐induced stress. Our analyses revealed several dose‐dependent changes in Raman peaks associated with carotenoid and phenylpropanoid abundance. We found these changes were specific to each HM, reflecting the activation of distinct stress‐response mechanisms. We also performed ICP‐MS of harvested rice tissue, allowing us to build Raman‐based calibration curves for predicting the HM concentration within rice. Lastly, we built a machine‐learning algorithm that could interpret the Raman spectra to diagnose the specific type of HM toxicity with an average of 84.5% accuracy after only 1 week of HM stress. These findings highlight the promise of RS as a valuable tool for real‐time, nondestructive monitoring of HM contamination in rice crops. Notably, the dose–response experimental design demonstrated RS's ability to detect HM stress levels that aligned with typical environmental contamination.

## 1 Introduction

As industrialization continues, contamination of farmland by toxic heavy metals (HMs) poses a significant threat to food security, especially in staple crops like rice. These soil‐borne HMs include arsenic, lead, mercury, cadmium, cesium, chromium, and nickel. Some HMs, notably arsenic and chromium, occur at high concentrations naturally in the environment, particularly in serpentine soils and areas with volcanic activity (Abdul Rashid et al. 2023; Yang et al. 2022). However, many of these pollutants result from current and past agricultural practices such as the application of organometallic pesticides and fertilizers (Rashid et al. 2023). Anthropogenic sources due to industrial and urban activities also contribute additional HMs (Adnan et al. 2022).

As such, studies of farmland and groundwater across the United States, Europe, and China consistently report elevated HM concentrations (Ren et al. 2022; Tóth et al. 2016; Zhou et al. 2020). Once introduced into the environment, HMs can readily bioaccumulate, especially within rice, which can accumulate arsenic at concentrations 10× to 20× higher than other grains (Liao et al. 2018; Nunes and Otero 2017). Given rice's role as a staple crop for half of the global population, a high level of HM exposure through daily rice consumption poses a serious public health risk (Angon et al. 2024). In major rice‐producing countries like China, India, and some Southeast Asian countries, contaminated rice contains HM levels deemed concerning to human health (Kumar et al. 2024; Mu et al. 2020; Ngo et al. 2024). Among these contaminants, arsenic, cadmium, and lead pose the greatest threat to human health, being ranked first, seventh, and second, respectively, on the ATSDR's substance priority list (Agency for Toxic Substances and Disease Registry 2017). Furthermore, the International Agency for Research on Cancer (IARC) classifies arsenic and cadmium as Group 1 carcinogens, while lead is a Group 2A probable carcinogen. All three are linked to cardiovascular and neurotoxic effects (Arruebarrena et al. 2023; Balali‐Mood et al. 2021; International Agency for Research on Cancer, n.d.; Solenkova et al. 2014). With over 520 million metric tons of rice being consumed annually, reducing HM contamination in rice is essential.

While long‐term solutions focus on mitigating HM uptake through bioengineering and soil remediation, current efforts focus on detecting and quantifying HM content in rice grains to reduce exposure risks. This is enabled by conventional analytical techniques such as inductively coupled plasma‐mass spectrometry (ICP‐MS), atomic absorption spectroscopy (AAS), and ion chromatography (IC) (Wang et al. 2022). Of these, ICP‐MS is the gold standard for detection of HMs within plant tissues due to its super low limit of detection and ability to simultaneously measure multiple analytes (Sader and Ryan 2020). However, its high cost, technical complexity, and requirement for destructive sample preparation limit accessibility for routine monitoring. Lower cost methods like AAS and IC also have limitations and still require destroying the plant tissue before analysis.

Recently, the Kurouski laboratory demonstrated that Raman spectroscopy (RS), a nondestructive analytical technique based on the inelastic scattering of light, could be used to detect arsenic exposure in rice by monitoring biochemical stress responses (Juárez et al. 2024). Specifically, arsenic‐induced oxidative stress led to carotenoid depletion and phenylpropanoid accumulation. Relative levels of these two classes of biomolecules could then be tracked using RS (Dou et al. 2021). It should be noted that Kurouski group also showed that RS could be used for the detection of a wide range of biotic and abiotic stresses in plants, including drought, nitrogen deficiency, and fundal and bacterial diseases (Farber et al. 2021; Farber et al. 2021; Sanchez et al. 2020; Sanchez et al. 2019; Sanchez et al. 2020; Morey et al. 2021). In all cases, the detection was based on unique changes in the chemical profile of plants that were detected using RS.

When Raman spectral data were correlated with ICP‐MS results, we found that RS could serve as a viable alternative for arsenic detection. However, this work used a high arsenic concentration of 50 μM, yet the World Health Organization's guideline for arsenic in drinking water is just 10 μg/L. This is the standard used by countries like the United States and China, while some developing nations permit up to 50 μg/L (Frisbie and Mitchell 2022; Uddin and Huda 2011). Arsenic is also rarely the sole contaminant in rice fields, with cadmium and lead posing greater risks in some regions. These metals often share similar toxicity mechanisms and trigger overlapping biochemical stress responses in plants, potentially confounding RS detection (Ghori et al. 2019; Huang et al. 2022). Therefore, two key questions remained: (1) How sensitive is RS to different levels of arsenic uptake, and (2) how specific is RS to arsenic stress compared to other HM exposures?

To address these gaps, we investigated the sensitivity and specificity of RS for diagnosing arsenic, cadmium, and lead uptake at three concentrations using a dose–response experimental design. Spectral data were analyzed using ANOVA and partial least squares discriminant analysis (PLS‐DA) to diagnose HM‐induced stress. HM accumulation was quantified using ICP‐MS, and Raman intensity at several peaks was correlated with metal content to produce calibration curves.

## 2 Methods/Materials

### 2.1 Experimental Design

**FIGURE 1:**
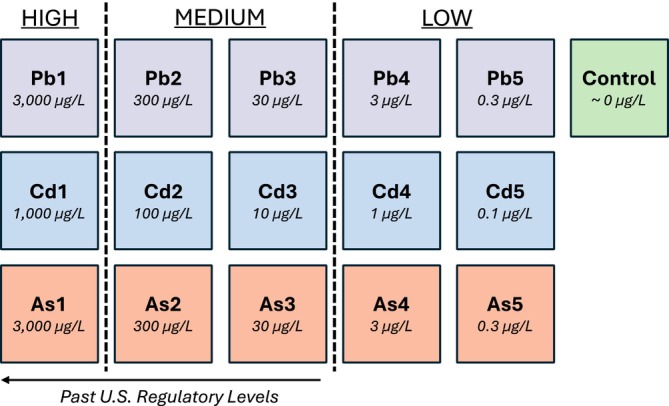
Schematic of experimental groups used in the HM dose–response experiment.

For the experiment, rice was cultivated in hydroponics using plastic containers and floated Styrofoam panels. Each panel had circular openings with plastic mesh underneath to support the roots of individual seedlings. The seeds were germinated before introduction to the system, with one seed per opening. The rice was nourished with a Yoshida nutrient solution, which included both macronutrients (114.30 mg/L NH_4_NO_3_, 50.40 mg/L NaH_2_PO_4_·2H_2_O, 89.30 mg/L K_2_SO_4_, 108.25 mg/L CaCl_2_ and 405 mg/L MgSO_4_·7H_2_O) and micronutrients (1.875 mg/L MnCl_2_·4H_2_O, 0.093 mg/L (NH_4_)_6_Mo_7_O_24_·4H_2_O, 1.09 mg/L H_3_BO_3_, 0.038 mg/L CuSO_4_·5H_2_O, 9.62 mg/L FeCl_3_·6H_2_O, 14.88 mg/L C_6_H_8_O_7_·H_2_O and 0.043 mg/L ZnSO_4_·7H_2_O). The water was changed out every 3 days, with nutrients added afterwards. The system was maintained at a pH of 5. The growth conditions were kept constant using a controlled chamber with a day/night cycle set to 12 h/12 h, humidity set to 55%, and day/night temperatures set to 29°C/26°C. After 2 weeks of growth, HM treatments were introduced. Plants were assigned to one of 16 groups, accounting for the combinations of experimental conditions and the control (Figure 1). HMs were administered concurrently with the Yoshida solution.

### 2.2 Instrumentation

An Agilent Resolve hand‐held Raman spectrophotometer was used to collect spectra from the rice leaves at 830 nm. Acquisition time was 1 s at a laser power of 495 mW; 24 Raman spectra were acquired for each group of plants once a week, stopping at Week 6. Photographs of the crops were also collected at these time points. All spectra were baselined and normalized at the 1440‐cm^−1^ peak.

Nitric acid digestions were performed to quantify the amount of heavy metal present in the rice tissue, following the procedure in Juárez et al. (2024). ICP‐MS was then run using a Quadrupole Inductively Coupled Plasma‐Mass Spectrometer (PerkinElmer NexION 300D) equipped with a Cetac ASX‐520 autosampler. Argon was used as the carrier gas. Rhodium was used as an internal standard. The calibration curve for ICP‐MS was generated using 1 g/L of certified reference material arsenic in 2% nitric acid. Dilutions of this external standard were made for 1, 25, 50, 100, and 200 ng/mL. All external standards and rice sample dilutions were made with ultrapure water.

### 2.3 Chemometrics

Microsoft Excel, R (programming language), and the PLS_toolbox (Eigenvector Research Inc.) were used in MATLAB to perform all statistical analyses and construct figures. Data were downloaded from the instrument as CSV (comma separated values) files and then imported into each software. ANOVA was performed in R for all peaks with visual change. 2D correlation spectra were generated from the averaged Raman spectra for each heavy metal. 3D surface plots were built to show the trend across time. PLS‐DA models were built for a binary comparison of each experimental group, with two to six latent variables used for each model. All data were checked for normality.

## 3 Results

**FIGURE 2:**
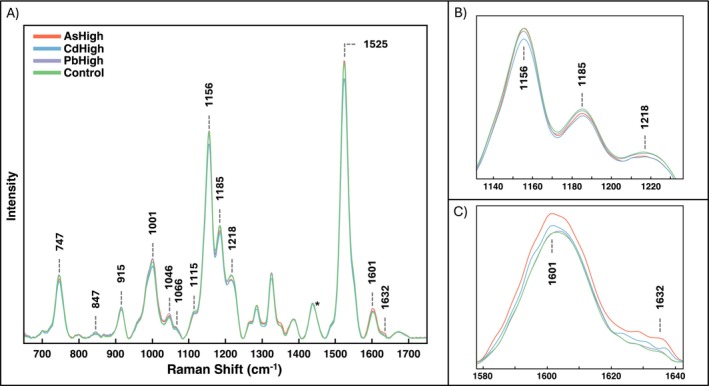
(A) Raman spectra collected from rice leaves of the experimental groups AsHigh, CdHigh, PbHigh, and the control at W6. Asterisk indicates the 1440‐cm^−1^ peak, used for normalization. (B) Zoomed in spectra of carotenoid peaks, and (C) zoomed in spectra of phenylpropanoid peaks.

**FIGURE 3:**
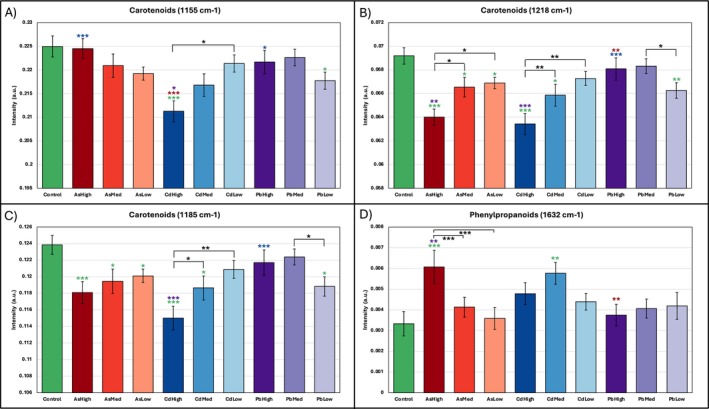
Bar plots of intensity at W6 at (A) 1155‐, (B) 1218‐, (C) 1185‐, and (D) 1632‐cm^−1^ peaks. *p ≤ 0.05, **p ≤ 0.01, and ***p ≤ 0.001. Green stars indicate significance against the control, while red, blue, and purple stars indicate significance versus the AsHigh, CdHigh, and PbHigh groups, respectively. Black stars indicate significance within the same HM condition.

The Raman spectra at Week 6 revealed many changes in peaks corresponding to different biomolecules (Figure 2). The most prominent spectral changes occurred at carotenoid (1155, 1185, 1218 cm^−1^), phenylpropanoid (1632 cm^−1^), nitrate (1046 cm^−1^), and amino acid (747 cm^−1^) peaks, along with smaller alterations in other peaks, including the other amino acid (915 cm^−1^) peak, carbohydrates (847 cm^−1^), cellulose (1115 cm^−1^), lipids (1066 cm^−1^), and additional carotenoid peaks (1001, 1525 cm^−1^) (Table S1). The largest decreases were observed at 1185 and 1218 cm^−1^ in the As and Cd groups, with an increase also at 1632 cm^−1^ caused by As. Bar graphs of the peak intensities confirmed that the change in carotenoid contents was dose dependent for the As and Cd groups, while the increase in phenylpropanoid content was dose dependent for the As group. Pb had no consistent dose‐dependent trend, and the peak intensities for PbHigh were closer to the control than PbLow (Figure 3).

The Kruskal–Wallis test revealed significant intensity changes occurred at 747‐, 1001‐, 1155‐, 1185‐, 1218‐, 1525‐, 1601‐, and 1632‐cm^−1^ peaks. CdHigh and AsHigh groups overall had the largest amount of significant peak changes relative to other experimental conditions (Figure S1). CdHigh spectra showed distinct decreases in the 1001, 1185 and 1218 cm^−1^ carotenoid peaks and the 747 cm^−1^ amino acid peak relative to the PbHigh group and control. Similarly, AsHigh spectra also decreased at 1185 and 1218 cm^−1^, but also showed significant increases at 1601 and 1632 cm^−1^ relative to the PbHigh group and control. Finally, AsHigh and CdHigh spectra could be distinguished by significant differences in intensity at the 1001‐, 1155‐, and 1525‐cm^−1^ carotenoid peaks. It should be noted that 1601‐ and 1632‐cm^−1^ peaks in all observed spectra were not symmetric, which indicates that a large group of aromatic compounds change in concentration in the case of HM toxicities. Additional mass‐spectroscopy analysis of such plants is necessary to fully understand the biochemical origin of HW‐induced toxicities in rice.

**FIGURE 4:**
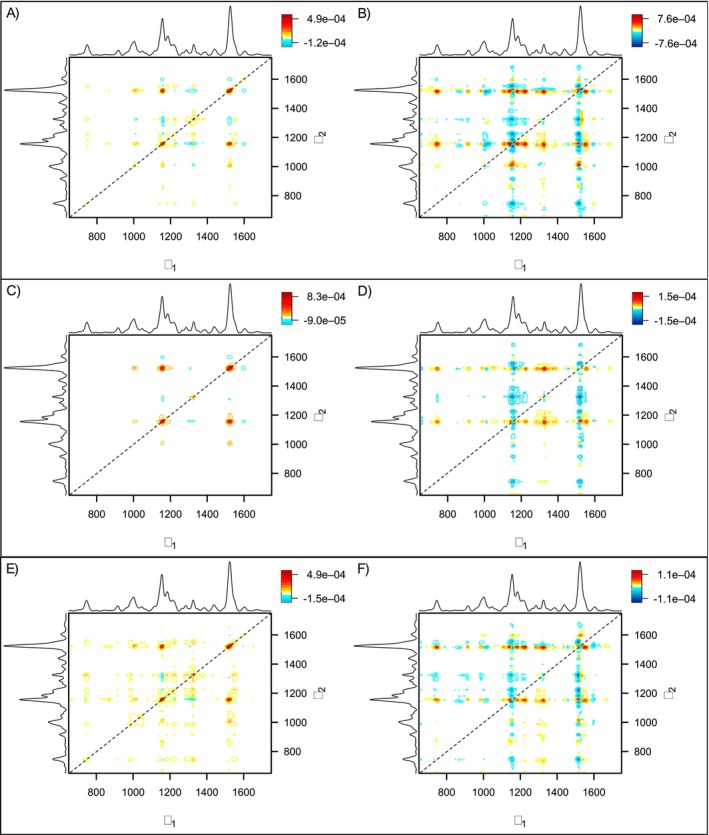
Two‐dimensional correlation spectroscopy (2D‐COS) analysis of the relationship between HM dosage and time. Plots (A, C, E) are the synchronous spectra for As, Cd, and Pb, respectively. Plots (B, D, F) are the asynchronous spectra for As, Cd, and Pb, respectively.

After statistical analysis, we performed 2D‐COS to extract spectral information from dosage‐dependent and time‐dependent perturbations (Figure 4). In the synchronous spectra of all three HMs, strong autopeaks (positive correlation peaks along the diagonal) at 1155 and 1525 cm^−1^ indicated these peaks undergo significant intensity alterations in response to HM stress. Smaller autopeaks were noted at the 1325 cm^−1^ aliphatic peak across all HM groups and at the 1632‐cm^−1^ peak in the As and Pb synchronous spectra. Additionally, two positive cross‐peaks at 1001–1525 cm^−1^ and 1155–1525 cm^−1^ were present in all synchronous spectra, indicating that intensity changes at these peaks occurred in the same direction.

The asynchronous spectra provided insights into the sequence of these linked spectral changes. Notably, the 1155‐cm^−1^ peak changed before the 1525‐cm^−1^ peak under As and Pb stress, whereas cadmium stress induced the opposite trend. In solely the As asynchronous spectrum, strong negative cross‐peaks at 1001–1155 and 1001–1525 cm^−1^ suggested that the 1001‐cm^−1^ peak decreases after the other two carotenoid peaks have decreased. Furthermore, peak splitting was observed at 1001 cm^−1^ for all three metals and at 1525 cm^−1^ for As and Pb, indicating a preferential degradation of certain carotenoid species.

**FIGURE 5:**
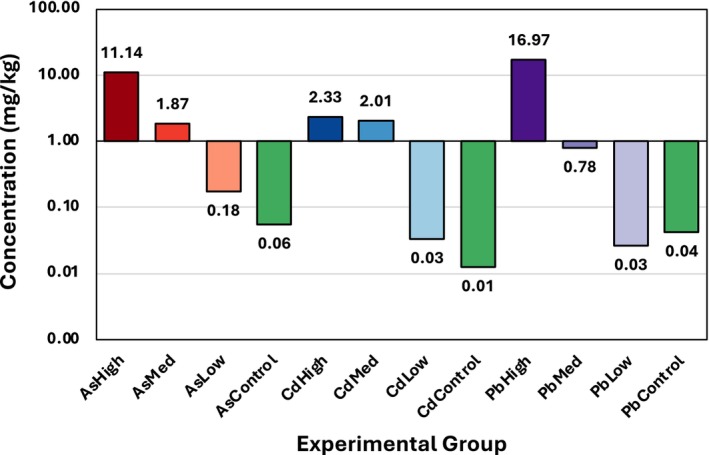
ICP‐MS results by experimental condition, indicating the average concentration of HM in rice leave.

To confirm that spectral findings reflected HM uptake, we performed ICP‐MS analysis of rice leaves harvested from Week 6. All experimental groups, except PbLow, had higher HM uptake than the control, with uptake increasing with higher HM dosages (Figure 5). Pb uptake exceeded As uptake despite an absence of observable symptoms or characteristic spectral changes. The amount of Cd taken up was similar between CdHigh and CdMed, while AsHigh and PbHigh had substantially elevated concentrations.

**FIGURE 6:**
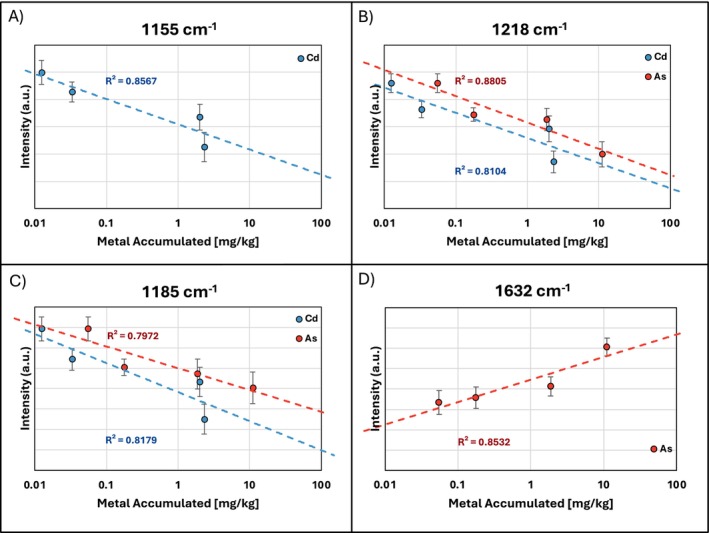
Calibration curves correlating Raman intensity with HM concentration at (A) 1155‐, (B) 1218‐, (C) 1185‐, and (D) 1632‐cm^−1^ peaks.

Correlating ICP‐MS values with Raman intensities enabled us to generate calibration curves for HM uptake. We generated four calibration curves for As and Cd using intensities from three carotenoid peaks (1155, 1185, 1218 cm^−1^) and one phenylpropanoid peak (1632 cm^−1^) (Figure 6). Most curves had high *r* ^2^ values, the strongest correlations being 0.8567 for Cd at the 1155‐cm^−1^ peak and 0.8805 for As at the 1218‐cm^−1^ peak. The 1632‐cm^−1^ peak also strongly correlated with As uptake but was the weakest correlation for Cd uptake. These results suggest that the 1155‐cm^−1^ peak is the best reference for assessing Cd uptake, while the 1218‐ and 1632‐cm^−1^ peaks together best assess As uptake. Notably, HM uptake followed a logarithmic rather than a linear relationship with intensity, contrary to our previous findings (Juárez et al. 2024). No Raman peaks correlated well with Pb uptake.

By instead correlating Raman intensities with the HM dosage in water, we generated dose–response curves. By then adding a temporal axis, we could generate 3D surface plots to visualize changes in peak intensity over time (Figure S2). By the first week of stress, the 1155‐ and 1185‐cm^−1^ peaks during Cd stress and the 1218‐cm^−1^ peak during As stress showed sharp declines in intensity with increasing dosage. In all three peaks, this trend persisted until week three, when lower‐dosage groups showed a partial recovery, suggesting an acclimation to HM levels. However, at Week 6, intensity continued to decline in AsHigh, AsMed, CdHigh, and CdMed groups. At the 1632‐cm^−1^ peak, As caused no major intensity changes until week three, when the AsHigh plants exhibited a sharp increase in phenylpropanoid content. By Week 6, a clear stress response was evident at the 1632‐cm^−1^ peak across all As dosages.

Lastly, we built a PLS‐DA model to evaluate the reliability of RS for diagnosing HM stress. Models were trained using Raman spectral data from Week 1 and Week 6 of HM stress. Binary PLS‐DA models, built comparing each group against the control and all other spectra within the same HM regardless of concentration, yielded strong classification performance (Table 1). Average prediction accuracies for As, Cd, and Pb were 84.5% at Week 1 and 82.3% at Week 6. While As and Pb accuracy were consistent at both time points, Cd had a higher true positive rate (TPR) at Week 1.

**TABLE 1:** PLS‐DA true positive rates for each experimental group at W1 and W6.

	Binary TPR		MCC	
Week	1	6	1	6
As1	0.896	0.892	0.776	0.775
As2	0.824	0.808	0.656	0.569
As3	0.840	0.783	0.680	0.644
As4	0.840	0.896	0.690	0.756
As5	0.783	0.840	0.585	0.683
As avg.	0.853	0.846	0.707	0.697
Cd1	0.912	0.766	0.817	0.595
Cd2	0.832	0.720	0.641	0.407
Cd3	0.784	0.725	0.602	0.446
Cd4	0.816	0.767	0.649	0.470
Cd5	0.904	0.808	0.785	0.594
Cd avg.	0.850	0.772	0.700	0.535
Pb1	0.864	0.783	0.713	0.594
Pb2	0.880	0.826	0.732	0.641
Pb3	0.808	0.942	0.657	0.818
Pb4	0.768	0.826	0.619	0.707
Pb5	0.824	0.950	0.585	0.917
Pb avg.	0.832	0.850	0.668	0.702
Control	0.933	0.859	0.859	0.757
Overall model avg.	0.845	0.823	0.692	0.645

## 4 Discussion

Across 6 weeks of HM stress, As1 rice developed substantially fewer tillers, while the As2 rice only had a slight reduction in tillers. The number of tillers appeared unaffected in the Cd1 rice; however, both the Cd1 and As1 rice had more dead leaves than other groups. Additionally, the younger leaves in the As1 rice had yellow‐green coloration, indicating mild chlorosis. All other experimental groups showed no visual symptoms (Figure S3). Also, despite the high HM levels noted for both arsenic and lead groups, HM levels decrease in plant tissue as they are translocated upwards, suggesting any grains grown from these rice plants would have lower concentrations of HMs than the leaves (Abedin et al. 2002).

Cd stress in rice could be characterized by tracking changes in multiple carotenoid peaks (1001, 1155, 1185, 1525 cm^−1^), as shown by both the average Raman spectra and results of Kruskal–Wallis. The 2D‐COS spectrum of Cd revealed that changes at the 1525‐cm^−1^ peak precede those at 1155 cm^−1^ and that the mechanism may be linked. The 1525‐cm^−1^ peak corresponds to C=C stretching, while the 1155‐cm^−1^ peak corresponds to asymmetric ring breathing. Under oxidative stress, carotenoids such as β‐carotene undergo oxidative cleavage into short‐chain volatiles, retaining the ring but losing double bonds (Havaux 2014; Ramel et al. 2012). This likely explains the earlier decrease in 1525 cm^−1^ intensity, which preceded degradation of the ring end‐group. 3D surface plots showed that intensities at 1155 and 1185 cm^−1^ naturally decreased as a function of rice age, regardless of cadmium dosage. The 1185‐cm^−1^ plot exhibited a sharp dip in week two, followed by partial recovery, suggesting a transient stress acclimation. This illustrates that carotenoid peaks can however change independently and must be assessed collectively to evaluate plant health. Both peaks correlated strongly with Cd uptake at Week 6.

As‐induced changes differed from Cd‐induced changes in two major ways. Notably, there was no significant decrease at the 1155‐cm^−1^ peak under high As levels, and there was a strong logarithmic increase in phenylpropanoids at 1632 cm^−1^. While the synchronous spectra for As and Cd were largely similar, the small autopeak at 1632 cm^−1^ was unique to arsenic. The asynchronous spectrum of As revealed a reversal in the order of carotenoid peak changes compared to Cd, with the 1155‐cm^−1^ peak instead changing before the 1525‐cm^−1^ peak. This further pointed to distinct stress response mechanisms between the two metals. Nevertheless, the changes at 1155 and 1525 cm^−1^ under arsenic stress were less consistent and not statistically significant at Week 6. The peaks at 1185 and 1218 cm^−1^ showed significant decreases with increasing As and Cd dosages. The 3D surface plot of the 1218‐cm^−1^ peak showed a sharp decrease in intensity at Week 2, followed by partial recovery in most groups, suggesting a transient stress acclimation similar to that observed under Cd stress at the 1185‐cm^−1^ peak. The 1218‐cm^−1^ peak also appeared as a small autopeak in the As synchronous map but not in Cd's, reinforcing a distinct carotenoid degradation pathway. The nature of the specific mechanism that could cause certain changes in carotenoid structure due to As stress but not Cd stress is unknown; however, differences in tested concentration between As and Cd may contribute to the observed divergence. In line with prior findings, phenylpropanoids at 1632 cm^−1^ remained the most consistent marker of As uptake. The 3D surface plot shows that phenylpropanoid accumulation in response to As stress is a delayed reaction in rice, initially limited to the AsHigh group until Week 6, when all As‐treated groups began producing phenylpropanoids at levels exceeding the control. Altogether, 1218‐ and 1632‐cm^−1^ peaks showed the strongest correlation with As uptake.

Pb‐induced spectral changes were highly erratic and lacked dose‐dependent trends. The absence of a clear relationship between intensity and dosage possibly suggests a more complex response to lead toxicity. As such, no reliable calibration curves could be generated linking Raman intensities to lead accumulation. Despite Pb‐induced spectral changes appearing less distinct, the PLS‐DA models readily differentiated Pb stress from other HMs‐induced stresses. This suggests that these subtle spectral alterations are sufficient for RS‐based machine learning models to reliably differentiate between the different HM stress patterns. Similar prediction rates across all metals further indicated that the lower statistical significance in Pb‐related spectral changes do not necessarily reflect a lack of a detectable physiological response.

## 5 Conclusion

Overall, these findings support the implementation of RS as a diagnostic method for arsenic, cadmium, and lead toxicity at a wide range of concentrations. The spectral changes linked to carotenoids were found to be particularly useful in assessing cadmium uptake, as were the carotenoid and phenylpropanoid peaks in assessing arsenic uptake. We found that the three HMs induced differential changes in carotenoid content, and that RS can utilize these differences in physiological markers to diagnose arsenic stress against other HMs. This work offers a much greater understanding of the limitations of RS and demonstrates its utility in detecting HM stress at concentrations relevant to typical environmental contamination. Looking forward, future studies should assess these findings within field conditions to further validate its implementation as a tool toward digital farming. Specifically, elucidation of the specificity of RS in general and observed changes in the concentrations of carotenoids and phenylpropanoids should be validated using a large number of HW and other similar chemicals.

## Conflicts of Interest

The authors declare no conflicts of interest.

## Peer Review

The peer review history for this article is available in the Supporting Information for this article.

## Supporting information


**Data S1** Supporting Information

**Table S1** Vibrational band assignment for Raman spectra collected from rice leaves.
**Figure S1.** Heatmap of Dunn’s post hoc test results sorted by peak and comparison.
**Figure S2.** 3D surface plot of HM dose–response in rice across six weeks. Maps were constructed for cadmium response at (A) 1155 cm^−1^ and (C) 1185 cm^−1^, and for arsenic response at (B) 1218 cm^−1^ and (D) 1632 cm^−1^. Red indicates a strong stress response as determined by Raman peak intensity.
**Figure S3.** Photographs of rice crops for each experimental condition at Week 6. The dosages for As and Pb start at 3000 μg/L (As1 and Pb1) and decrease logarithmatically for each group (ex. As5 = 0.3 μg/L). The dosages for Cd start at 1000 μg/L (Cd1) and decrease logarithmically for each group (ex. Cd5 = 0.1 μg/L). Control was not given any HM dosage.
