# Supplementary material for: Specificity and Selectivity of Raman Spectroscopy for the Detection of Dose‐Dependent Heavy Metal Toxicities
Source: Plant Direct. 2025 Jun 23;9(6):e70086. doi: 10.1002/pld3.70086 (PMC12185781; doi:10.1002/pld3.70086)
Supplement: Supplementary file 2 — Table S1 Vibrational band assignment for Raman spectra collected from rice leaves. Figure S1. Heatmap of Dunn’s post hoc test results sorted by peak and comparison. Figure S2. 3D surface plot of HM dose–response in rice across six weeks. Maps were constructed for cadmium response at (A) 1155 cm−1 and (C) 1185 cm−1, and for arsenic response at (B) 1218 cm−1 and (D) 1632 cm−1. Red indicates a strong stress response as determined by Raman peak intensity. Figure S3. Photographs of rice crops for each experimental condition at Week 6. The dosages for As and Pb start at 3000 μg/L (As1 and Pb1) and decrease logarithmatically for each group (ex. As5 = 0.3 μg/L). The dosages for Cd start at 1000 μg/L (Cd1) and decrease logarithmically for each group (ex. Cd5 = 0.1 μg/L). Control was not given any HM dosage. [file PLD3-9-e70086-s002.pdf]

# Specificity and Selectivity of Raman Spectroscopy for the Detection of Dose-dependent Heavy Metal Toxicities

*Isaac D. Juárez[1,2], Nicholas Shepard[3], Cole Sebok[4], Sudip Biswas[5], Endang Septiningsih[5], Dmitry Kurouski[1,2]**

1. Department of Biochemistry and Biophysics, Texas A&M University, College Station, Texas 77843, United States
2. Interdisciplinary Faculty of Toxicology, Texas A&M University, College Station, Texas 77843, United States
3. Interdisciplinary Graduate Program in Molecular & Environmental Sciences, Texas A&M University, College Station, Texas 77843, United States
4. Department of Biology, Texas A&M University, College Station, Texas 77843, United States
5. Department of Soil and Crop Sciences, Texas A&M University, College Station, Texas 77843, United States

## SUPPLEMENTARY FIGURES

**Table S1.** Vibrational band assignment for Raman spectra collected from rice leaves.

| **Peak ($cm^{-1}$)** | **Vibration** | **Assignment** |
|---|---|---|
| 747 | γ(C-O-H) of R-$CO_2$H | Amino Acids |
| 847 | Skeletal mode of (C-O-C) | Carbohydrates |
| 915 | ν(C-O-C) | Cellulose/Proline |
| 1001 | Ring breathing of Phe | Carotenoids |
| 1046 | ν(C-O) + ν(C-C) + δ(C-O-H) | Nitrates |
| 1066 | ν(C-C) | Lipids |
| 1115 | $ν_s$(C-O-C) + δ(C-O-H) | Cellulose |
| 1155 | ν(C-O-C) + ν(C-C) + asymmetric ring breathing | Carotenoids |
| 1185 | ν(C-O-H) next to aromatic ring + σ bond (C-H) | Carotenoids |
| 1218 | δ(C-C-H) | Carotenoids |
| 1525 | ν(C=C) | Carotenoids |
| 1601 | Aromatic ring ν(C-C) | Phenylpropanoids |
| 1632 | Aromatic ring ν(C=C, C=O) | Phenylpropanoids |

| | 747 | 847 | 915 | 1001 | 1046 | 1066 | 1115 | 1155 | 1185 | 1218 | 1525 | 1601 | 1632 |
|---|---|---|---|---|---|---|---|---|---|---|---|---|---|
| Con – AsHigh | 0.059 | 0.471 | 0.066 | 0.110 | 0.228 | 0.903 | 0.028 | 0.942 | 0.008 | 0.001 | 0.425 | 0.007 | 0.001 |
| Con – AsMed | 0.129 | 0.017 | 0.676 | 0.736 | 0.256 | 0.181 | 0.036 | 0.156 | 0.019 | 0.016 | 0.926 | 0.888 | 0.321 |
| Con – AsLow | 0.233 | 0.060 | 0.286 | 0.404 | 0.805 | 0.702 | 0.069 | 0.089 | 0.037 | 0.036 | 0.627 | 0.467 | 0.918 |
| Con – CdHigh | 0.001 | 0.052 | 0.025 | 0.042 | 0.446 | 0.215 | 0.064 | 0.001 | 0.001 | 0.001 | 0.005 | 0.261 | 0.055 |
| Con – CdMed | 0.503 | 0.101 | 0.456 | 0.843 | 0.527 | 0.715 | 0.235 | 0.077 | 0.035 | 0.021 | 0.379 | 0.031 | 0.002 |
| Con – CdLow | 0.687 | 0.004 | 0.587 | 0.344 | 0.017 | 0.497 | 0.103 | 0.312 | 0.117 | 0.061 | 0.412 | 0.571 | 0.091 |
| Con – PbHigh | 0.388 | 0.973 | 0.068 | 0.315 | 0.102 | 0.377 | 0.414 | 0.430 | 0.397 | 0.422 | 0.895 | 0.919 | 0.591 |
| Con – PbMed | 0.670 | 0.098 | 0.781 | 0.700 | 0.105 | 0.514 | 0.609 | 0.474 | 0.406 | 0.405 | 0.496 | 0.811 | 0.352 |
| Con – PbLow | 0.296 | 0.013 | 0.498 | 0.861 | 0.236 | 0.726 | 0.050 | 0.036 | 0.013 | 0.009 | 0.495 | 0.298 | 0.625 |
| AsHigh – AsMed | 0.507 | 0.113 | 0.085 | 0.128 | 0.799 | 0.225 | 0.655 | 0.127 | 0.467 | 0.033 | 0.403 | 0.003 | 0.001 |
| AsHigh – AsLow | 0.320 | 0.284 | 0.288 | 0.007 | 0.248 | 0.595 | 0.465 | 0.070 | 0.324 | 0.014 | 0.154 | 0.001 | 0.001 |
| AsMed – AsLow | 0.685 | 0.529 | 0.421 | 0.146 | 0.270 | 0.033 | 0.729 | 0.728 | 0.751 | 0.696 | 0.471 | 0.281 | 0.269 |
| CdHigh – CdMed | 0.001 | 0.543 | 0.062 | 0.029 | 0.125 | 0.279 | 0.333 | 0.053 | 0.019 | 0.006 | 0.015 | 0.383 | 0.354 |
| CdHigh – CdLow | 0.001 | 0.528 | 0.002 | 0.001 | 0.001 | 0.033 | 0.614 | 0.007 | 0.004 | 0.002 | 0.001 | 0.463 | 0.604 |
| CdMed – CdLow | 0.183 | 0.129 | 0.110 | 0.154 | 0.029 | 0.194 | 0.574 | 0.354 | 0.507 | 0.609 | 0.035 | 0.049 | 0.077 |
| PbHigh – PbMed | 0.568 | 0.102 | 0.066 | 0.437 | 0.794 | 0.715 | 0.666 | 0.848 | 0.888 | 0.929 | 0.591 | 0.719 | 0.749 |
| PbHigh – PbLow | 0.039 | 0.013 | 0.005 | 0.178 | 0.484 | 0.502 | 0.301 | 0.226 | 0.127 | 0.087 | 0.398 | 0.241 | 0.898 |
| PbMed – PbLow | 0.070 | 0.303 | 0.238 | 0.490 | 0.593 | 0.710 | 0.075 | 0.088 | 0.043 | 0.029 | 0.092 | 0.323 | 0.586 |
| AsHigh – CdHigh | 0.069 | 0.218 | 0.685 | 0.001 | 0.047 | 0.168 | 0.723 | 0.001 | 0.235 | 0.671 | 0.001 | 0.113 | 0.087 |
| AsHigh – PbHigh | 0.005 | 0.488 | 0.001 | 0.548 | 0.663 | 0.442 | 0.161 | 0.383 | 0.067 | 0.002 | 0.501 | 0.009 | 0.002 |
| CdHigh – PbHigh | 0.001 | 0.054 | 0.001 | 0.002 | 0.015 | 0.032 | 0.294 | 0.015 | 0.003 | 0.001 | 0.003 | 0.301 | 0.162 |

| | |
|---|---|
| *NS* | *>0.05* |
| * | *≤0.05* |
| ** | *≤0.01* |
| *** | *≤0.001* |

**Figure S1.** Heatmap of Dunn's post-hoc test results sorted by peak and comparison.

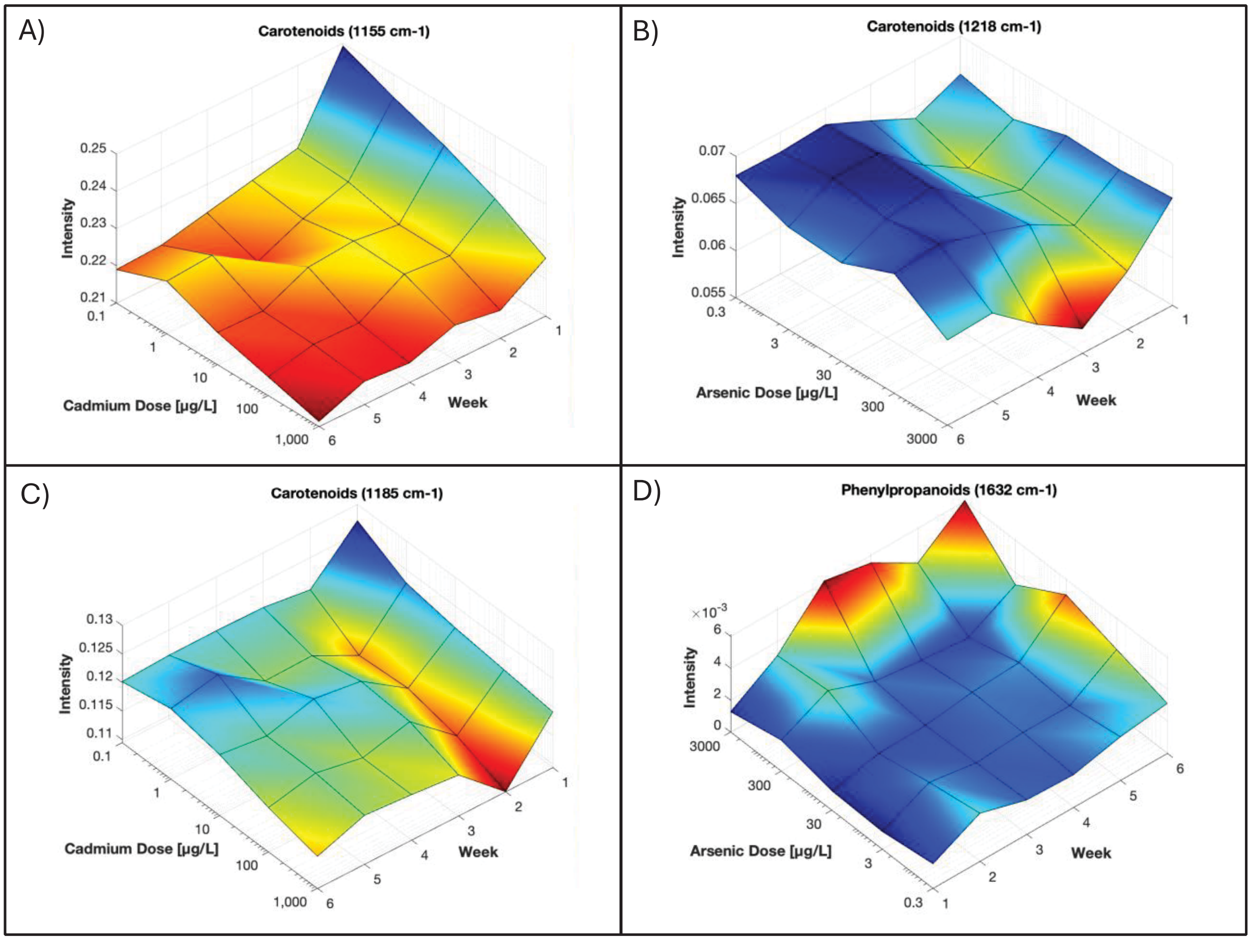


**Figure S2.** 3D surface plot of HM dose-response in rice across six weeks. Maps were constructed for cadmium response at (A) 1155 $cm^{-1}$ and (C) 1185 $cm^{-1}$, and for arsenic response at (B) 1218 $cm^{-1}$ and (D) 1632 $cm^{-1}$. Red indicates a strong stress response as determined by Raman peak intensity.

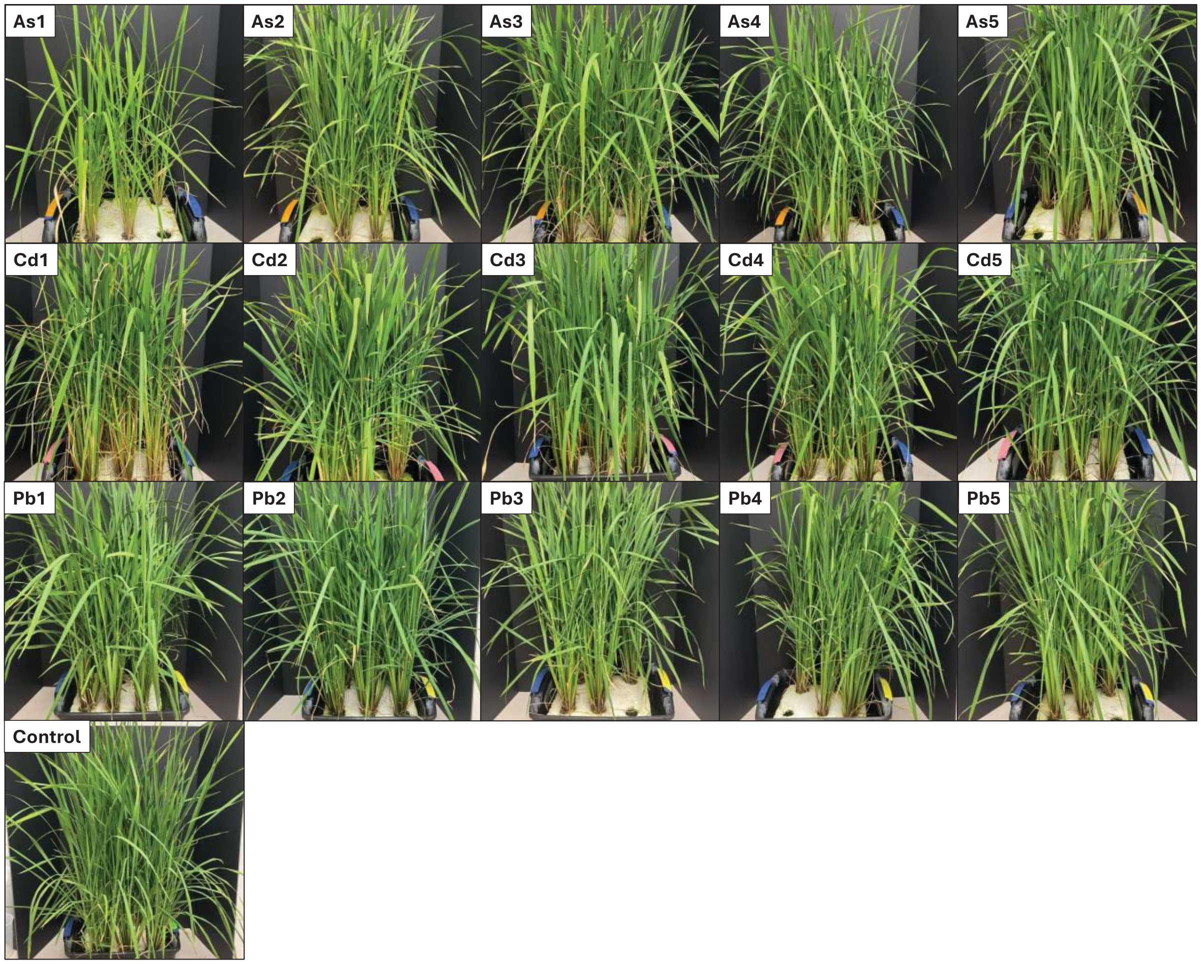


**Figure S3.** Photographs of rice crops for each experimental condition at Week 6. The dosages for As and Pb start at 3,000 µg/L (As1 and Pb1) and decrease logarithmically for each group (ex. As5 = 0.3 µg/L). The dosages for Cd start at 1,000 µg/L (Cd1) and decrease logarithmically for each group (ex. Cd5 = 0.1 µg/L). Control was not given any HM dosage.
