# Supplementary material for: Specificity and Selectivity of Raman Spectroscopy for the Detection of Dose‐Dependent Heavy Metal Toxicities
Source: Plant Direct. 2025 Jun 23;9(6):e70086. doi: 10.1002/pld3.70086 (PMC12185781; doi:10.1002/pld3.70086)
Supplement: Supplementary file 1 — Data S1 Supporting Information [file PLD3-9-e70086-s001.pdf]

Plant Direct
Open Access

Specificity and Selectivity of Raman Spectroscopy for the Detection of Dose-dependent Heavy Metal Toxicities

Prof. Dmitry Kurouski , Mr. Isaac Juarez , Mr. Nicholas Shepard , Mr. Cole Sebok , Endang M. Septiningsih

Decision letter round 1

June 10, 2025

Prof. Dmitry Kurouski

Texas A&M University

Biochemistry and Biophysics

300 Olsen Blvd

Department of Biochemistry and Biophysics

College Station, Texas 77843

RE: Specificity and Selectivity of Raman Spectroscopy for the Detection of Dose-dependent Heavy Metal Toxicities

Dear Dr. Kurouski:

Thank you for submitting to Plant Direct. All required reviews have been returned and we have now finished our evaluation of your manuscript. In light of the reviewers' and editor's comments, further revisions are needed before the paper can be accepted for publication in Plant Direct.

Please view the editors' and reviewers' comments below and use their suggestions as a guide while you work on your revision.

When uploading the revised version of this article, please be sure to include the following:

-A word document that contains your response to the reviewers. You should respond to each reviewer comment and note the changes made to the manuscript. If you do not agree with a reviewer's comment and choose not to make a suggested revision, please explain why. Please try to provide as complete an answer as possible to each reviewer's criticisms.

-A tracked changes document with each change highlighted

- A clean version of the latest version of the manuscript

To upload your revision, please click the link below.

https://plantdirect.msubmit.net/cgi-bin/main.plex?el=A2Lr2ou7A5nAH1I2A9ftdoIEOEelYJCT0zIFWf4ZmJwZ

In order to provide as timely a service as possible, we ask that your revision is resubmitted within three months after receipt of this request. If an extension is needed, please send a request, along with a brief explanation, to the editorial office at plantdirect@wiley.com .

Please note that, in addition to publishing reviewer comments, the author's responses to review comments will also be published alongside the final version of the paper. If you would not like the author's responses to be published, please contact the editorial office at plantdirect@wiley.com .

Thank you very much for giving us an opportunity to review your work. I look forward to receiving the next version.

Sincerely,

Hannah Schneider

Esther van der Knaap

Plant Direct
Open Access

Editor, Plant Direct

-- Editor comments:

-- Reviewer comments:

Reviewer #1:

In the reported study, Juarez and co-workers investigated the potential of Raman spectroscopy (RS) for the detection of heavy metal stresses in rice. Using a dose-response experimental design, the researchers modelled various levels of arsenic, cadmium, and lead-induced stress in plants. The researchers observed dose-dependent changes in Raman peaks associated with carotenoid and phenylpropanoid abundance. Juarez and co-workers found specific changes in the spectra associated with each metal, reflecting the activation of distinct stress-response mechanisms. The researchers also performed ICP-MS of harvested rice tissue and built Raman-based calibration curves for predicting the heavy-metal concentrations within rice.

Overall, the reported findings highlight the promise of RS as a valuable tool for real-time, non-destructive monitoring of heavy metal contamination in rice crops. Notably, the dose-response experimental design demonstrated RS's ability to detect HM stress levels that aligned with typical environmental contamination. Overall, all experiments were designed and performed well, as well as thoroughly documented. The researchers used advanced statistical methods for data analysis, which makes the reported results sound and reliable.

What needs to be addressed:

The introduction could benefit from more in-depth discussion of previously reported papers where RS was used.

The peak around 1600 cm-1 is not symmetric which indicates that changes in phenolic profiles of plants are rather complex. Can the researcher comment on this observation?

Once these two minor issues are addressed, the paper can be accepted for the publication.

Reviewer #2:

The manuscript „Specificity and Selectivity of Raman Spectroscopy for the Detection of Dose-dependent Heavy Metal Toxcities" was submitted to Plant Direct for publication.

Broad comments:

The study describes the heavy metal-induced changes of phenylpropanoids, carotenoids and other organic compounds by RAMAN spectroscopy.

The experimental work was well described, and the introduction give enough background to introduce the reader into the work. Also, the results are clearly presented and properly discussed, at least regarding the changes in signals related to the compound concentrations.

However, in my opinion idea of indirectly estimating heavy metal concentrations falls to short. There are also other phenomena except from the three investigated heavy metals that could pose stress to plants and thus lead to a shift in specific compound concentrations. This can either be other heavy metals or organic contaminants as well as herbivores which will cause a reaction of the plants.

Therefore, the conclusion of the work to correlate carotenoids with cadmium and phenylpropanoids with arsenic is not only not supported but also a bit dangerous as many other potential factors are ignored.

Hence, additional factors must be investigated and tested, or the general idea of the manuscript must be reconsidered refraining from "substituting" techniques such as ICP, AAS or IC, which directly quantify the amount of heavy metals.

Decision letter round 2

June 11, 2025

Prof. Dmitry Kurouski

Texas A&M University

Biochemistry and Biophysics

300 Olsen Blvd

Department of Biochemistry and Biophysics

Plant Direct

Open Access

College Station, Texas 77843

MSID: 2025-01807R1

MS TITLE: Specificity and Selectivity of Raman Spectroscopy for the Detection of Dose-dependent Heavy Metal Toxicities

Dear Dr. Dmitry Kurouski:

I am pleased to inform you that your manuscript "Specificity and Selectivity of Raman Spectroscopy for the Detection of Dose-dependent Heavy Metal Toxicities" has been accepted for publication in Plant Direct.

Your article will appear online in the next available issue of Plant Direct. To ensure your article gets published as quickly as possible, please pay attention to the steps detailed below. We have found that most of the delays happen at this stage, especially at the payment stage, so please respond as quickly as possible when prompted.

License Agreement: Once your article has been accepted it will move to Production and undergo admin and file checking - you may receive an email with any queries we have at this stage. When all required items are received by the publisher and queries resolved, the corresponding author will receive an email from Wiley's Author Services system which will ask them to log on at https://authorservices.wiley.com/bauthor and will present them with the appropriate license for completion. Your article cannot be published until both the signed license agreement and payment of the article fee have been received.

Payment of the Open Access Article Publication Fee: All articles published in Plant Direct are fully open access: immediately and freely available to read, download and share. Plant Direct charges a publication fee to cover the publication costs. The corresponding author for this manuscript should have already received a quote from the payments team (cs-openaccess@wiley.com) with the estimated article publication fee; please email cs-openaccess@wiley.com if this has not been received. The corresponding author should log on to the Wiley Author Services site, where the publication fee can be paid by credit card or an invoice. Pro Forma can also be requested. Payment of the publication charge must be received before the article will be published online.

Plant Direct
Open Access

Proofs: You will have the opportunity to look over your paper once more when you receive the author proofs for your article. The proofs will be with you in approximately two weeks. Please note that, in addition to publishing reviewer comments, the author's responses to review comments will also be published alongside the final version of the paper. If you would not like the author's responses to be published, please contact the editorial office at plantdirect@wiley.com .

Promotion of your article: You can help your research get the attention it deserves! Check out Wiley's free Promotion Guide for best-practice recommendations for promoting your work at www.wileyauthors.com/eeo/guide. And learn more about Wiley Editing Services which offers professional video, design, and writing services to create shareable video abstracts, infographics, conference posters, lay summaries, and research news stories for your research at www.wileyauthors.com/eeo/promotion.

Thank you again for your contribution to Plant Direct. If you have any questions, feel free to contact the editorial office at plantdirect@wiley.com .

Sincerely,

Hannah Schneider

Esther van der Knaap

Editor, Plant Direct

Response to reviewer round 1

Reviewer 1:

The introduction could benefit from more in-depth discussion of previously reported papers where RS was used.

Response: We are grateful to the authors for the provided suggestion. To address it, we added the following sentences:

“Relative levels of these two classes of biomolecules could then be tracked using RS (Dou et al., 2021). It should be noted that Kurouski group also showed that RS could

be used for the detection of a wide range of biotic and abiotic stresses in plants, including drought, nitrogen deficiency, fundal and bacterial diseases (Farber et al., 2021a, Farber et al., 2021b, Sanchez et al., 2020a, Sanchez et al., 2019, Sanchez et al., 2020b, Morey et al., 2021). In all cases, the detection was based on unique changes in the chemical profile of plants that were detected using RS."

The peak around 1600 cm-1 is not symmetric which indicates that changes in phenolic profiles of plants are rather complex. Can the researcher comment on this observation?

Response: We are grateful to the authors for the provided suggestion. To address it, we added the following sentences:

"It should be noted that 1601 and 1632 cm-1
peaks in all observed spectra were not symmetric,
which indicates that a large group of aromatic compounds change in concentration in the case of HM toxicities. Additional mass-spectroscopy analysis of such plants is necessary to fully understand the biochemical origin of HW-induced toxicities in rice."

Reviewer #2:

However, in my opinion idea of indirectly estimating heavy metal concentrations falls to short. There are also other phenomena except from the three investigated heavy metals that could pose stress to plants and thus lead to a shift in specific compound concentrations. This can either be other heavy metals or organic contaminants as well as herbivores which will cause a reaction of the plants.

Therefore, the conclusion of the work to correlate carotenoids with cadmium and phenylpropanoids with arsenic is not only not supported but also a bit dangerous as many other potential factors are ignored.

Hence, additional factors must be investigated and tested, or the general idea of the manuscript must be reconsidered refraining from "substituting" techniques such as ICP, AAS or IC, which directly quantify the amount of heavy metals.

Response: We agree with the reviewer. Therefore, we modified the conclusion section of the manuscript:

"Overall, these findings support the implementation of RS as a diagnostic method for

arsenic, cadmium, and lead toxicity at a wide range of concentrations."

And added the following sentence at the end of the manuscript:

"Specifically, elucidation of specificity of RS in general and observed changes in

the concentrations of carotenoids and phenylpropanoids should be validated using a large

number of HW and other similar chemicals."
